# Phosphoinositide Profile of the Mouse Retina

**DOI:** 10.3390/cells9061417

**Published:** 2020-06-07

**Authors:** Stella Finkelstein, Sidney M. Gospe, Kai Schuhmann, Andrej Shevchenko, Vadim Y. Arshavsky, Ekaterina S. Lobanova

**Affiliations:** 1 Department of Ophthalmology, Duke University, Durham, NC 27710, USA; stella@duke.edu (S.F.); sid.gospe@duke.edu (S.M.G.III); vadim.arshavsky@duke.edu (V.Y.A.); 2 Max Planck Institute of Molecular Cell Biology and Genetics, 01307 Dresden, Germany; schuhman@mpi-cbg.de (K.S.); shevchenko@mpi-cbg.de (A.S.); 3 Department of Pharmacology and Cancer Biology, Duke University, Durham, NC 27710, USA; 4 Department of Ophthalmology, University of Florida, Gainesville, FL 32610, USA; 5 Department of Pharmacology and Therapeutics, University of Florida, Gainesville, FL 32610, USA

**Keywords:** retina, phosphatidylinositol phosphate, phosphatidylinositol bisphosphate, photoreceptor

## Abstract

**** Phosphoinositides are known to play multiple roles in eukaryotic cells. Although dysregulation of phosphoinositide metabolism in the retina has been reported to cause visual dysfunction in animal models and human patients, our understanding of the phosphoinositide composition of the retina is limited. Here, we report a characterization of the phosphoinositide profile of the mouse retina and an analysis of the subcellular localization of major phosphorylated phosphoinositide forms in light-sensitive photoreceptor neurons. Using chromatography of deacylated phosphatidylinositol headgroups, we established PI(4,5)P_2_ and PI(4)P as two major phosphorylated phosphoinositides in the retina. Using high-resolution mass spectrometry, we revealed 18:0/20:4 and 16:0/20:4 as major fatty-acyl chains of retinal phosphoinositides. Finally, analysis of fluorescent phosphoinositide sensors in rod photoreceptors demonstrated distinct subcellular distribution patterns of major phosphoinositides. The PI(4,5)P_2_ reporter was enriched in the inner segments and synapses, but was barely detected in the light-sensitive outer segments. The PI(4)P reporter was mostly found in the outer and inner segments and the areas around nuclei, but to a lesser degree in the synaptic region. These findings provide support for future mechanistic studies defining the biological significance of major mono- (PI(4)P) and bisphosphate (PI(4,5)P_2_) phosphatidylinositols in photoreceptor biology and retinal health.

## 1. Introduction

Phosphoinositide phospholipids are essential components of membranes in every cell. The parent phosphatidylinositol (PI) can be phosphorylated combinatorially on the inositol ring at the 3-, 4-, or 5- position(s). Levels of phosphorylated phosphatidylinositols are tightly controlled by the activities of specific phosphoinositide kinases and phosphatases. Individual forms of phosphorylated phosphoinositides serve as unique signals to engage a variety of phosphoinositide binding proteins, thus affecting diverse cellular processes, including protein transport, cell polarity, cytoskeletal organization, ion-channel function, and gene expression [1,2,3,4]. Dysregulation of phosphoinositide metabolism has been reported to result in retinal dysfunction in animal models and humans. For instance, zebrafish mutants deficient in SynJ1, a phosphoinositide phosphatase, demonstrate signs of disrupted transport of synaptic proteins and defects in autophagosomal and endosomal trafficking within photoreceptors [5,6]. The loss of PI-3 kinase Vps34 in mice leads to deficiencies in endosome recycling and aggressive degeneration of photoreceptors [7]. Genetic inactivation of the p110α-subunit of PI-3 kinase in cones similarly demonstrated the critical role of PI3K signaling in photoreceptor survival [8]. In humans, mutations in the phosphoinositide metabolizing enzyme INPP5E have been linked to progressive retinopathy in Joubert syndrome [9,10], and abnormalities in phosphoinositide-binding proteins have been linked to photoreceptor degeneration in humans and mice [11,12,13]. The phosphoinositide binding proteins have been proposed to play a role in vesicular transport in rods [14,15]. Finally, several publications indicate that light regulates phosphoinositide metabolism in the retina (reviewed in [16,17]). However, the functional significance of this regulation is unknown, and the direction of light-induced phosphoinositide changes remains controversial.

Despite accumulating evidence that phosphoinositide signaling plays a critical role in many cellular functions, little is currently known about the phosphoinositide composition of the retina. Historically, phosphoinositides were studied in cell culture using radionuclide labeling approaches. However, the poor viability of the retina in vitro limits the utility of radiolabeling-based approaches in analyzing this tissue. Furthermore, these methods allow monitoring of phosphoinositide turnover, but cannot measure absolute phosphoinositide levels. Therefore, in this study, we took advantage of modern non-radiolabeling techniques to profile phosphorylated phosphoinositides of the mouse retina. Our study establishes PI(4)P and PI(4,5)P_2_ as major phosphorylated headgroups and 16:0/20:4 and 18:0/20:4 as dominant fatty acid species of retinal phosphatidylinositols. Furthermore, our analysis identifies distinct patterns of subcellular localization of these lipids within photoreceptors. These findings position PI(4)P and PI(4,5)P_2_ lipids as central players in future studies of phosphoinositide metabolism in photoreceptors. 

## 2. Materials and Methods

### 2.1. Animals

All procedures conformed to the Association for Research in Vision and Ophthalmology Statement for the Use of Animals in Ophthalmic and Vision Research and were approved by the Institutional Animal Care and Use Committee of University of Florida (study # 201709934, approved 9/20/2017). Wild type C57BL/6J mice were purchased from Jackson Laboratory (stock # 000664) and housed under a 12/12 h dark/light cycle. Mice were maintained on a plant-based diet (Teklad global 18% protein rodent diet #2918, Envigo). For dark adaptation, mice were kept in a dark room for 12 h. For light conditioning, dark-adapted mice with dilated pupils were exposed to a light intensity of 500 lux for one hour. Animals were euthanized with isoflurane, followed by decapitation after the animals were rendered non-responsive. For lipid analysis, retinas were carefully dissected from enucleated eyes under infrared (for dark adaptation experiments) or room light (for light adaptation experiments) in Ringer’s solution, flash-frozen in liquid nitrogen, and stored at -80 °C. For subcellular localization studies of phosphoinositide sensors, the retinal agarose sections were prepared as previously described [18,19]. Briefly, eyes were fixed overnight in 4% paraformaldehyde prepared in phosphate-buffered Saline (PBS), rinsed in PBS, and embedded in 4% low-melt agarose. 100-µm retinal sections were collected using a Leica vibratome (VT1200S), mounted on slides under coverslips with Fluoromount (Electron Microscopy Sciences), and analyzed with a Nikon A1 Spectral Confocal Microscope.

### 2.2. Chemicals

Chemicals and solvents of ACS (American Chemical Society) or LC–MS (Liquid Chromatography–Mass Spectrometry) grade were purchased from Sigma-Aldrich, and synthetic lipid standards were purchased from Avanti Polar Lipids. 

### 2.3. Phosphatidylinositol Extraction and Detection with Electroconductivity Method

Phosphatidylinositol extraction for HPLC chromatography was performed using a one-step acidified Folch method. It is generally accepted that due to their negative charge, phosphorylated phosphatidylinositols remain tightly bound to proteins and cannot be extracted using standard lipid extraction procedures. However, protonation of phosphoinositides with the addition of hydrochloric acid (HCl) neutralizes the negative charge on phosphoinositides, allowing their efficient extraction from proteins and partitioning from aqueous into organic phase during a standard Folch procedure. Flash-frozen retinas were homogenized in an ice-cold chloroform/methanol mixture (2C:1M supplemented with 0.1% of HCl) in the presence of 200 μL of acid-washed beads (G4649; Sigma) by bead beating (Mini-Bead-Beater-24, Biospec). Organic and aqueous phases were separated by the addition of 1*M* HCl and centrifugation at 5000× *g* for 5 min at 4 °C. Lipids were collected from the lower organic phase, dried under a stream of nitrogen, and deacylated as described [20]. Briefly, the lipid pellet was resuspended in 200 µl monomethylamine (40%): butanol: methanol: water (36:9:47:8), incubated in a Thermomixer (Eppendorf) at 53 °C for 50 min, chilled and dried in a SpeedVac (Eppendorf). The pellet was resuspended in 100 μL water, and fatty acids were extracted twice with 100 μL N-butanol: petroleum ether: ethyl formate (20:4:1). The aqueous phase containing headgroups of anionic phospholipids was dried in a SpeedVac, resuspended in 75 μL H_2_O, and stored at −20 °C.

Delipidated headgroups were separated on the Ionpac AS11-HC column (and AG11-HC guard column) connected to the ICS-2500 Dionex HPLC system. The column was equilibrated first with 10 mM NaOH and then with 1.5 mM NaOH. After sample injection (25 μL), elution was carried out in four stages at a flow rate of 0.38 mL/min: (1) gradient from 1.5 mM to 4 mM NaOH for 7 min; (2) 4 to 16 mM NaOH for 5 min; (3) 16 to 86 mM NaOH for 18 min; (4) 15 min isocratic elution with 86 mM NaOH. Elution was monitored by suppressed conductivity detection with the ED50 electrochemical detector. Commercially available phosphorylated phosphatidylinositols (Avanti Polar Lipids) were used for phosphoinositide profiling after deacylation. For experiments requiring quantification and calibration, concentrations of PI(4)P and PI(4,5)P_2_ standards were measured by the colorimetric determination of phosphorus after digestion to inorganic phosphate and taking into consideration the number of phosphorus atoms in the corresponding lipid [21].

### 2.4. Phosphatidylinositol Extraction and Detection with Mass Spectrometry

In preliminary studies, we established that due to low abundance and ion suppression, phosphorylated phosphoinositides could not be detected with shotgun lipidomics in crude lipid extracts prepared by the one-step acidified Folch method described above. Therefore, we implemented a two-step procedure allowing preparation of retinal extract enriched for phosphorylated phosphatidylinositols, but significantly depleted of other phospholipids as described [22]. Extracted lipids were dried under a vacuum, resuspended in methanol supplemented with 0.1% HCl, infused into an LTQ orbitrap mass spectrometer (Thermo Scientific) with Triversa NanoMate (Advion), and analyzed in negative ion mode. Lipids were identified and quantified by LipidXplorer software [23]. Quantifications and calibrations were performed using lipidomics grade standards PI(4)P 17:0/20:4 and PI(4,5) P_2_ 17:0/20:4, (Avanti Polar Lipids), which are not present naturally in the mouse retina and have distinct and non-overlapping mass spectra compared to endogenous phosphoinositides. Phosphorylated phosphoinositides were extracted in the presence of standards containing approximately 1/5th of tissue homogenate of one mouse retina. Phosphatidylinositol profiling of retina was performed as described in [24]. The profiling of fatty acid composition of major mouse phosphatidylinositols (PI) was performed at Lipotype (Dresden, Germany). Phosphatidylinositol species were annotated using the total number of carbon atoms and the total number of double bonds in fatty acid moieties combined or individually. For example, phosphatidylinositol lipid containing 18:0 (stearic) and 20:4 (arachidonic) fatty acids was abbreviated as PI 38:4 and PI 18:0/20:4.

### 2.5. Distribution of PI(4)P and PI(4,5)P_2_ in Rod Photoreceptors

Genes of interest were subcloned into a plasmid driving expression under the 2.2 kb bovine rhodopsin promoter [25]. Constructs included rhodopsin C-terminally tagged with mCherry, and Green Fluorescent Protein (GFP)-tagged phosphoinositide binding domains from phospholipase Cδ1 (PLCδ1), Tubby, and four-phosphate-adaptor protein 1 (FAPP1) proteins as PI(4,5)P_2_ and PI(4)P sensors [26,27,28,29]. Constructs coding for sensors were delivered into photoreceptors by in vivo electroporation, as described in [25]. Briefly, neonatal mice (P0.5) were anesthetized on ice, and their sclera punctured through the eyelid with a 30-gauge needle at the six o’clock position. A solution containing 0.5 μL of 5–6 μg DNA mix was delivered subretinally with a blunt-end 32-gauge needle attached to a Hamilton Syringe. DNA solution contained vectors coding for rhodopsin-mCherry and phosphoinositide sensor in equal amounts. Electroporation was performed by delivering five 100 V pulses of 50 ms duration with ECM830 pulse generator (BTX, Harvard Apparatus) using tweezer electrodes covered with Gonak solution (Akorn) and positioned over the mouse eye. After electroporation, mice were returned to the cages and analyzed six weeks later. Three to five successfully electroporated animals were examined for each experimental condition. 

### 2.6. Statistical Analysis

Statistical analysis, including linear regression fits, was carried out using Graph Pad PRISM 8. Data are expressed as means +/- standard deviation. Moreover, *p*-values below 0.05 (*p* < 0.05) were considered statistically significant.

## 3. Results

### 3.1. Retinal Composition of Major Phosphoinositide Headgroups

**Figure 1:**
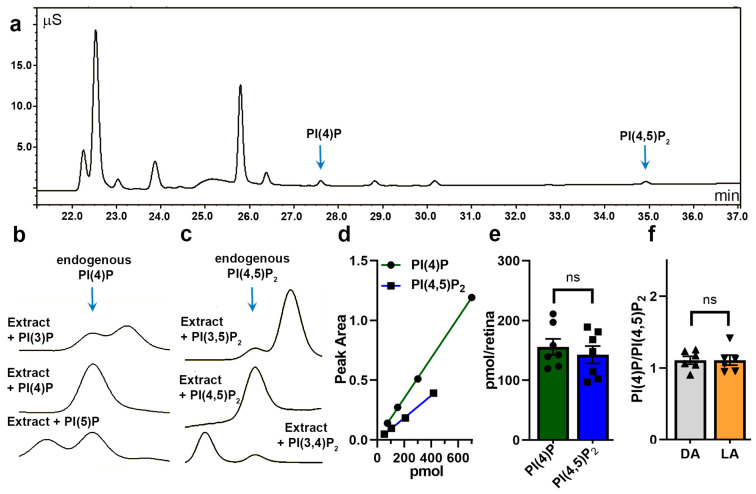
PI(4)P and PI(4,5)P_2_ are the major phosphorylated phosphoinositides in the mouse retina. (**a**) Representative chromatogram of headgroups from delipidated phosphoinositides extracted from the mouse retina, separated by anion-exchange HPLC and monitored by suppressed conductivity detection. (**b**,**c**) Chromatograms of delipidated headgroups obtained from mouse retinal extracts separated in the presence of different delipidated phosphorylated phosphoinositide standards. PI(4) (**b**) and PI(4,5)P_2_ (**c**) are major phosphorylated inositide headgroups in the retina. (**d**) Calibration curves for headgroups from delipidated PI(4)P and PI(4,5)P_2_ standards are linear in the range 50-800 pmol (n = 3; error bars are not plotted due to SD values being smaller than symbol sizes). (**e**) The retina contains comparable amounts of PI(4)P and PI(4,5)P_2_ (~150 pmol; *p* = 0.52). (**f**) The PI(4)P to PI(4,5)P_2_ ratio in the retina remains unchanged in dark-adapted (DA, gray) and light-adapted (LA, orange) mice (*p* = 0.98).

To establish a profile of phosphorylated phosphoinositides in the mouse retina, we took advantage of a previously reported method allowing the separation of deacylated phosphoinositide headgroups by anion exchange high-pressure liquid chromatography (HPLC) and detection of eluted ions by suppressed conductivity [20]. A typical HPLC separation profile of delipidated headgroups obtained from mouse retina is shown in Figure 1a. The two peaks eluted at characteristic retention times of ~27.5 and ~35 min correspond to PI(4)P and PI(4,5)P_2_, respectively (Figure 1a, arrows). The identity of each peak was established by mixing deacylated retinal extracts from mouse and deacylated standards (Figure 1b). In the example shown in Figure 1b, supplementing the retinal extract with deacylated PI(3)P and PI(5)P resulted in the appearance of additional peaks, whereas the addition of PI(4)P increased the height of the original PIP peak, indicating that this peak represented PI(4)P. The same approach was used to establish the identity of the PIP_2_ peak as PI(4,5)P_2_ (Figure 1c).

Next, using deacylated PI(4)P and PI(4,5)P_2_ standards, we established the linearity of this method in a range from 50 to 800 pmol (Figure 1d). We noted that the slope for PI(4,5)P_2_ is more shallow than for PI(4)P, so an identical amount of PI(4,5)P_2_ yields a smaller peak than PI(4)P. This is most likely due to a higher negative charge of PI(4,5)P_2_, which requires higher concentrations of NaOH for elution and a higher extent of ion suppression for detection. In pilot studies, we found significant variability in the handling of phosphoinositides under 25 pmol; therefore, we aimed our subsequent measurements at around 100 pmol. The absolute amounts of PI(4)P and PI(4,5)P_2_ extracted from one mouse retina under optimized extraction and delipidation protocols approached ~150 pmol per retina (Figure 1e). This method requires substantial sample handling and does not allow consistent control of material loss during extraction and delipidation, limiting our ability to reliably assess potential light-dependent changes of the total pools of phosphoinositides. Therefore, we instead analyzed changes in the ratio of PI(4)P/PI(4,5)P_2_ extracted from retinas of dark- and light-adapted mice (Figure 1f). These measurements did not reveal any statistically significant light-dependent phosphoinositide changes.

### 3.2. Fatty Acid Composition of Major Retinal Phosphoinositides

One limitation of the conductivity detection method is a loss of information about the fatty acid composition of PI(4)P and PI(4,5)P_2_ upon their delipidation. Therefore, we complemented phosphoinositide profiling with shotgun lipidomics and high-resolution mass spectrometry. This method allows the identification of lipid species by accurate mass measurements and the identification of the acyl composition of phospholipids by fragmentation.

**Figure 2:**
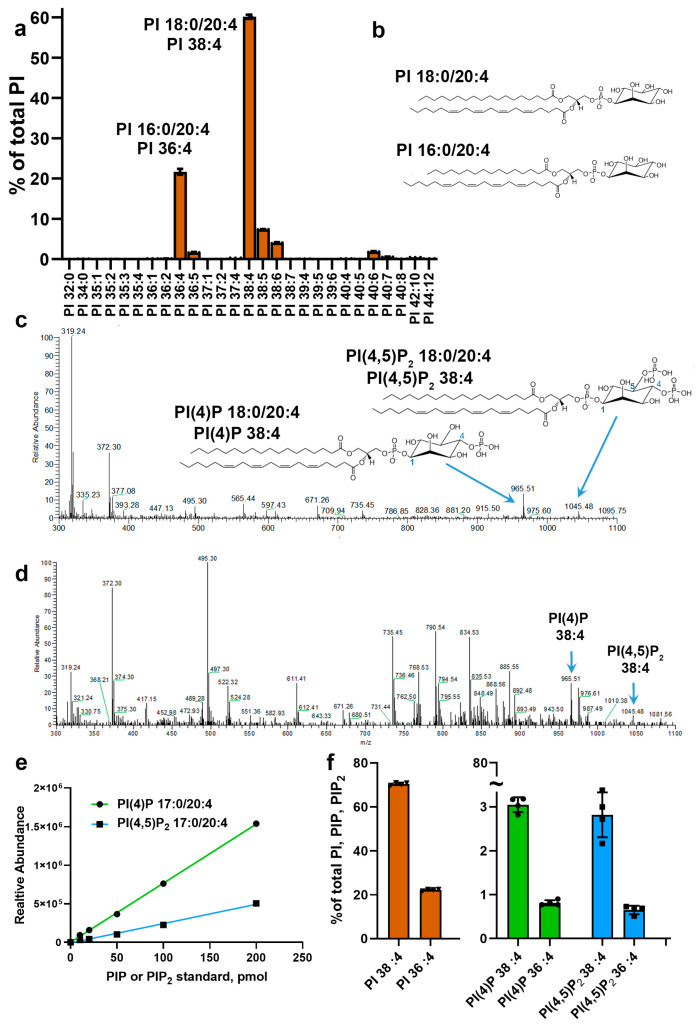
Lipidomic analysis of retinal phosphoinositides. (**a**) Lipid profiles of retinal phosphatidylinositols in 5-week-old wild type mice (mean+/-SD, n = 3). Phosphatidylinositols are shown as a percentage of the total. (**b**) Structures of major arachidonic acid-containing PI species, 16:0/20:4 and 18:0/20:4. (**c**) Mass spectra and structures of PI(4)P 16:0/20:4 and PI(4,5)P_2_ 18:0/20:4 standards detected by direct infusion with high-resolution mass spectrometry as single ions in negative mode (blue arrows). (**d**) Representative mass spectra of lipids from mouse retinal extracts collected in negative ion mode. Peaks representing endogenous PI(4)P 38:4 and PI(4,5)P_2_ 38:4 are marked with blue arrows. (**e**) Calibration curves of PI(4)P 17:0/20:4 and PI(4,5)P_2_ 17:0/20:4 standards are linear in the 10 to 200 pmol range (n = 3; error bars are not plotted due to SD values being smaller than symbol sizes). (**f**) Lipid profiles of 36:4 and 38:4 phosphatidylinositols (PI, brown) and their phosphorylated forms (PI(4)P, green; PI(4,5)P_2_, blue) in dark-adapted mouse retinas (mean+/-SD, n = 4).

In the first set of experiments, we looked at the fatty acid composition of retinal phosphatidylinositols, the precursors of phosphorylated phosphoinositides. The phosphatidylinositol profile was dominated by two species containing arachidonic acid: PI 18:0/20:4 (PI 38:4) and PI 16:0/20:4 (PI 36:4) (Figure 2a; see Figure 2b for illustrations of their structures). These accounted for ~60% and ~22% of the total retinal PI, respectively. Notably, we also detected trace amounts of PI species containing fatty acids with odd carbon numbers. It is generally accepted that mammals do not synthesize fatty acids with an odd number of carbons de novo. Therefore, these fatty acids are most likely obtained from a plant-based food diet [30,31].

In control experiments employing phosphorylated forms of the predominant retinal phosphatidylinositols PI(4)P 18:0/20:4 (PIP 38:4) and PI(4,5)P_2_ 18:0/20:4 (PIP_2_ 38:4) as standards, we confirmed that these species are detected as single ions in negative mode (Figure 2c). This is consistent with previous lipidomic studies of phosphoinositides in other tissues [32]. Note that other peaks in Figure 2c represent a typical nonspecific spectral background and correspond to chemicals extracted from laboratory plastics exposed to organic solutions during sample preparation and infused into the mass spectrometer. In a separate set of experiments, we established an approach allowing us to detect the ions representing endogenous mono- and bisphosphate inositides in mouse retinal extracts (Figure 2d, marked with blue arrows). This analysis, combined with the results of the electroconductivity studies described above, suggests that these ions correspond to PI(4)P 38:4 and PI(4,5)P_2_ 38:4. Next, using as standards phosphorylated phosphatidylinositols that are not naturally present in mammalian membranes (PI(4)P 37:4 and PI(4,5)P_2_ 37:4), we established the linearity of this method and a detection limit as low as 10 pmol (Figure 2e).

Our analysis of the phosphoinositide composition of the mouse retina is summarized in Figure 2f. First, we established the dominant phosphorylated phosphatidylinositols as 38:4 and 36:4 acylated species. Second, we found that the retina contains comparable amounts of PI(4)P (green) and PI(4,5)P_2_ (blue), confirming the results of electroconductivity studies described above. Third, we estimated that phosphorylated inositides account for ~7% of total phosphatidylinositols containing 38:4/36:4 acyls. Finally, we observed that the ratio of 38:4 to 36:4 acyl species is essentially unaffected by the status of inositol phosphorylation.

### 3.3. Subcellular Distribution of PI(4,5)P_2_ and PI(4)P in Mouse Rod Photoreceptors

In the final set of experiments, we investigated the localization of phosphoinositides in rod photoreceptors. This was accomplished by delivering constructs encoding GFP sensors of PI(4,5)P_2_ and PI(4)P into mouse rods by in vivo electroporation. Previous studies had reported light-dependent changes in the levels of these phosphoinositides [33] and their modulatory effects on the phototransduction cascade [34] and enzymatic activities (also reviewed in [16,17,35,36]). Therefore, we designed experiments allowing us to assess the presence of phosphoinositides in different subcellular compartments of the rod cells, particularly the light-sensitive outer segment. To this end, DNA constructs encoding the phosphoinositide sensors were electroporated together with fluorescently tagged rhodopsin, a resident protein of outer segments.

**Figure 3:**
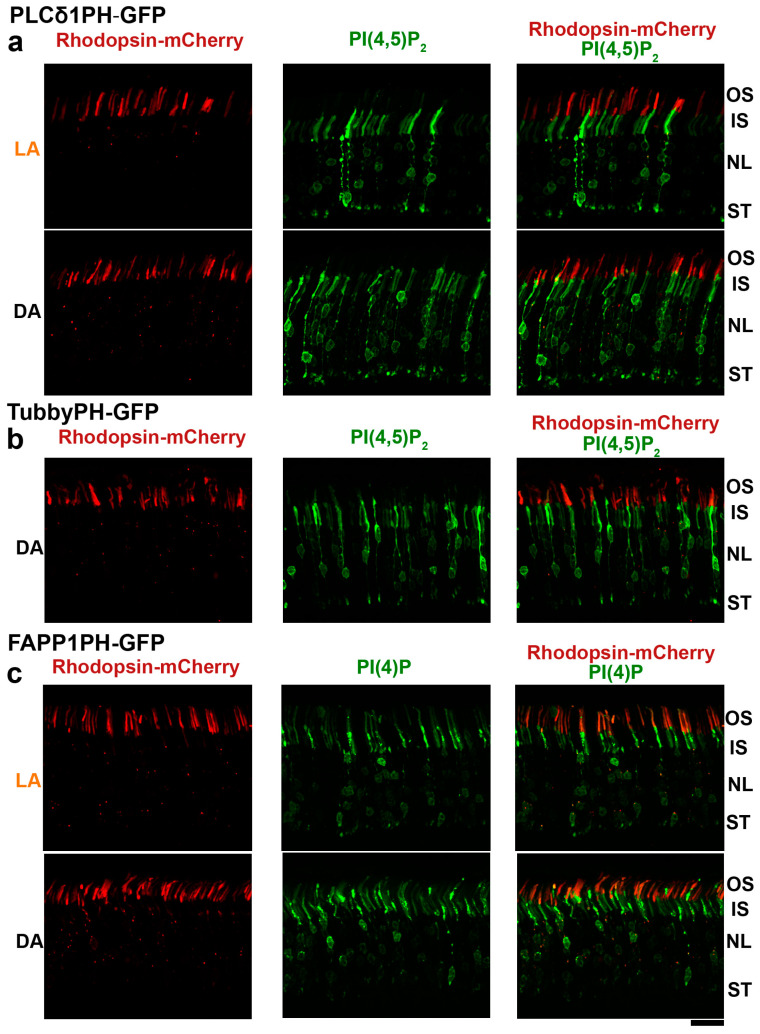
Distribution of PI(4)P and PI(4,5)P_2_ phosphoinositide sensors in rod photoreceptors. Green Fluorescent Protein (GFP) fusion constructs of PI(4,5)P_2_-binding domains from PLC δ1 (**a**) and Tubby (**b**) proteins and the PI(4)P-binding domain of FAPP1 (**c**) were electroporated at P0 into rod photoreceptors to investigate the localization of corresponding phosphoinositides. Their distribution patterns (green) were analyzed in retinal cross-sections of 6-week-old dark (DA)- or light (LA)- adapted mice. Rhodopsin fused with mCherry (red) was used as a rod outer segment marker. PI(4,5)P_2_ sensors produce intense signals in the inner segments (IS), nuclear layer (NL) and synaptic terminals (ST), but are virtually excluded from outer segments (OS). The PI(4)P sensor shows signal in the outer segments, around nuclei, and in the inner segments. Occasional red puncta detected in the inner segments most likely represent mistargeted Rhodopsin-mCherry fusion protein. Scale bar: 20 μm.

The most commonly used sensor of PI(4,5)P_2_, PLCδ1PH-GFP, was found mostly outside ofouter segments, with an intense labeling of the inner segments and synapses (Figure 3a). Localization of this sensor in rods of light- and dark-adapted retinas had a similar pattern. The same localization pattern was observed for TubbyPH-GFP, an alternative specific sensor of PI(4,5)P_2_ (Figure 3b). In contrast, the FAPP1PH-GFP sensor of PI(4)P (Figure 3c) was present in the outer segments, as illustrated by its overlapping localization with mCherry-tagged rhodopsin. We also observed a sizable fraction of PI(4)P sensor in the inner segments and in an area around the nuclei. Overall, there was no definitive change in the localization patterns of the PI(4)P sensor in dark- and light-adapted mice. The localization pattern of the sensors in relation to the outer segments was consistent with a previous analysis, which documented the presence of PI(4)P but not PI(4,5)P_2_ in preparations of bovine outer segments [20].

## 4. Discussion

Studies conducted over the past decade have highlighted the complexity of cellular processes controlled by phosphorylated forms of phosphoinositides, including cytoskeleton organization, channel modulation, endosomal and lysosomal transport, protein trafficking, cell polarization, and gene expression. However, the functional significance of phosphoinositide metabolism in the retina is poorly understood, and the phosphoinositide composition of this tissue has remained uncharacterized. Traditionally used radiolabeling techniques may assess turnover of lipids and changes in enzymatic activities but do not allow measurement of the absolute levels of individual phosphoinositides. Until recently, progress in this direction was hindered by a lack of sensitive techniques suitable for phosphoinositide quantification without lipid radiolabeling. In this study, we used modern non-radioactive analytical methods to characterize the composition of phosphorylated phosphatidylinositols in the mouse retina.

Using chromatography of delipidated phosphoinositide headgroups, we have established P(4,5)P_2_ and P(4)P as the major phosphorylated phosphatidylinositols in the mouse retina. The levels of PI(4,5)P_2_ and PI(4)P are comparable, and a mouse retina contains approximately 150 pmol of each lipid. This number most likely represents a low-end estimate due to less than 100% efficiency of lipid extraction from the tissue.

Several reports suggest that light regulates the levels of PI(4)P and PI(4,5)P_2_ in rod outer segments (reviewed in [16]). However, the biological function of this regulation remains unknown, and even the direction of the light-induced phosphoinositide changes was not consistent across these studies. Early studies with radioactive labeling suggested that the PI(4,5)P_2_ level in whole retinas is reduced by light [37] and implicated the activation of phospholipase C [38]. In contrast, another study from the same laboratory detected light-stimulated synthesis of PI(4)P and PI(4,5)P_2_ in isolated rod outer segments in vitro [39]. Unfortunately, the electroconductivity method we utilized requires extensive sample handling, which does not allow control of material loss during extraction and delipidation, limiting our ability to reliably assess potential light-dependent changes of the total pools of phosphoinositides in different samples. Therefore, we instead compared ratios of PI(4)P/PI(4,5)P_2_ extracted from retinas of the dark- and light-adapted mice. This analysis did not reveal statistically significant changes, indicating either that both lipids change proportionally or that they do not change at all. However, we cannot exclude the possibility that analyzing whole retina extracts might mask small changes in the profiles of these lipids within individual subcellular compartments and/or subsets of retinal cells.

The contents of other phosphoinositides, PI(3)P, PI(5)P, PI(3,4)P_2_, PI(3,5)P_2,_ PI(3,4,5)P_3_, were below our detection limit, indicating that their retinal content lies somewhere below single picomolar levels. Although they were not detected here, they are most likely present in the retina and may play significant roles in retinal health. Consistent with this notion, inactivation of enzymes handling PI(3)P was reported to result in defects in mouse photoreceptors and bipolar cells [7,8,40]. Future development and validation of sensitive analytical methods allowing measurement of phosphorylated phosphoinositides at femtomolar levels, similar to the recently reported ELISA-based approach [7], will be necessary to accurately quantify these less abundant species.

Using mass spectrometry-based lipidomics, we found that at least 82% of the total phosphatidylinositide pool in the mouse retina is represented by arachidonic acid-containing species, PI 38:4, and PI 36:4. The same acyl composition was identified for mono- and bisphosphate phosphorylated forms (PIP 36:4/38:4 and PIP_2_ 36:4/38:4), which account for ~7% of total retinal phosphatidylinositol. The predominance of arachidonic acid-containing PI(4)P and PI(4,5)P_2_ species is largely consistent with phosphoinositide profiles of mammalian membranes and neuronal tissues [41,42]. However, the biological significance of selective enrichment of phosphatidylinositols with arachidonic acid remains unknown [43]. 

Finally, we used genetic phosphoinositide sensors to infer the subcellular location of PI(4,5)P_2_ and PI(4)P within rod photoreceptors. The main difference in the subcellular distribution of these phosphoinositide species is that PI(4)P was present in the light-sensitive outer segment, whereas PI(4,5)P_2_ was mostly excluded from this compartment. This observation is consistent with a previous study, which reported the presence of PI(4)P but not PI(4,5)P_2_ in membranes of bovine outer segments [20]. We also noted significant enrichment of the PI(4,5)P_2_ sensor but not the PI(4)P sensor in photoreceptor synapses. The latter is consistent with zebrafish studies demonstrating the critical role of SynJ1 (a phosphatase regulating the levels of 5-phosphate-containing phosphoinositides) in the synapses of photoreceptors [5,6]. Admittedly, any interpretation of phosphoinositide distributions based on localization patterns of sensors should be conservative, due to the inherent limitations of a protein overexpression approach [44,45]. Sensor overexpression could disrupt the distribution of endogenous phosphoinositides and phosphoinositide-binding proteins and is subject to variability among individual photoreceptors and injection procedures. Nevertheless, the stark dissimilarity in the sensors’ distributions is encouraging and supports the notion of distinct subcellular localization patterns of PI(4)P and PI(4,5)P_2_ in rods. Notably, our overexpression experiments did not reveal dramatic rearrangements of sensors under dark/light conditions. However, we cannot exclude the possibility that this approach is not sensitive enough to detect small light-dependent rearrangements. The development of stable transgenic mice expressing sensors at controlled levels and recent advances in high-resolution microscopy will permit a more rigorous assessment of the role of PI(4)P and PI(4,5)P_2_ as key players in inositide signaling in the retina.

## 5. Conclusions

Phosphatidylinositol can be phosphorylated combinatorially on three positions, producing seven distinct forms of phosphoinositides. Our study establishes PI(4)P and PI(4,5)P_2_ as predominant phosphoinositide species in the mouse retina and describes subcellular distribution patterns of these lipids in rod photoreceptors. Combined with reports linking mutations in enzymes controlling levels of phosphoinositides phosphorylated at the fifth position with retinal disorders in humans [9,10,46], these findings encourage further mechanistic studies to define precise functional roles of PI(4)P and PI(4,5)P_2_ lipids in the retina. 

The goal of future studies is to establish which phosphoinositide kinases and phosphatases are critical for maintaining the levels of PI(4)P and PI(4,5)P_2_ in this tissue and, most importantly in photoreceptors. It would also be interesting to use nonradioactive methods of phosphoinositide detection to compare the phosphoinositide composition of mouse and human retinas. Another future goal is to address whether pathological changes in the levels and distribution of phosphoinositides contribute to blindness in diseases that are not directly caused by disruptions of phosphoinositide metabolism, such as retinitis pigmentosa or syndromic ciliopathies [47,48,49,50,51,52].

## Abbreviations

C	chloroform
DA	dark-adapted
FAPP1	four-phosphate-adaptor protein 1
GFP	green fluorescent protein
HCl	hydrochloric acid
HPLC	high-performance liquid chromatography
INPP5E	inositol polyphosphate-5-phosphatase E
LA	light-adapted
M	methanol
NaOH	sodium hydroxide
PBS	phosphate-buffered saline
PH	pleckstrin homology domain
PI	phosphatidylinositol
PI(4)P	phosphatidylinositol 4-monophosphate
PI(4,5)P_2_	phosphatidylinositol 4,5-bisphosphate
PI3K	phosphoinositide 3-kinase
PLCδ	phospholipase Cδ
ROS	rod outer segments
SynJ1	synaptojanin 1
Vps34	vacuolar protein sorting 34
